# Oncostatin M for Anti-HBV Therapy: Can a Foe Be Turned Into a Friend?

**DOI:** 10.1016/j.jcmgh.2023.11.005

**Published:** 2023-11-25

**Authors:** Tao Cheng, Yun Cheng, Dong-Yan Jin

**Affiliations:** School of Biomedical Sciences, The University of Hong Kong, Pokfulam, Hong Kong; State Key Laboratory of Liver Research, The University of Hong Kong, Pokfulam, Hong Kong

Hepatitis B virus (HBV) chronically infects more than 290 million people worldwide, and, among them, liver cirrhosis or hepatocellular carcinoma eventually develops in a large subset.^1^ Vaccines are highly effective. Antivirals based on nucleoside analogs and interferon α (IFN-α) potently reduce viral loads and hepatic inflammation. However, HBV hardly can be eradicated in chronically infected patients owing to the persistence of covalently closed circular DNA (cccDNA). Even a functional cure defined as serologic recovery with normal liver function, absence of hepatitis B surface antigen, and undetectable HBV DNA can be achieved in only a small subset (< 8%) of infected people under antiviral treatment.^2^ Because most functionally cured people have minimal or no clinical consequences, achieving this status is the current goal of anti-HBV therapy. Direct-acting antivirals and host-targeting agents are 2 main categories of new anti-HBV pharmaceuticals under intense investigations and development. Some recent highlights of new anti-HBV agents being developed include ARB-1467, GSK3228836, and GS9620.^3^, ^4^, ^5^ ARB-1467 is a small interfering RNA that leads to the cleavage of HBV messenger RNAs.^3^ GSK3228836 is an antisense oligonucleotide that also targets HBV messenger RNAs.^4^ GS9620 is an innate immune activator that enhances host antiviral immunity.^5^

In this issue of *Cellular and Molecular Gastroenterology and Hepatology*, Ye et al^6^ screened interleukin (IL)6 family cytokines for anti-HBV potency and found that oncostatin M (OSM) strongly suppresses HBV replication. In patients with HBV chronic infection, serum OSM concentrations were dampened and correlated inversely with hepatitis B surface antigen and hepatitis B e antigen levels. Transcriptomic analysis indicated that OSM activated JAK-STAT signaling to induce the expression of gene products with functions in innate and adaptive immunity, including IFITMs, S100A9, LCN2, and IL1R. The requirement of 1 OSM-induced protein, interferon-induced transmembrane protein 1 (IFITM1), for OSM-mediated anti-HBV activity was further shown in HepG2.2.15 and HBV-infected HepG2-NTCP cells. Consistent with the in vitro results, OSM promoted immune clearance of HBV in a mouse model of persistent HBV replication.^6^

IL6 family cytokines are involved in metabolism, inflammation, and liver regeneration by engaging several signaling pathways such as JAK-STAT, mitogen-activated protein kinase, and phosphoinositide 3 kinase-Akt. IL6 family cytokines play an important role in host defense against bacterial, viral, and fungal infections.^7^ For instance, IL6 inhibits taurocholic acid cotransport polypeptide (NTCP)-mediated viral entry and hepatocyte nuclear factor 4α–dependent cccDNA transcription to repress HBV replication.^8^ In addition, OSM inhibits the replication of hepatitis C virus in Huh7 cells.^9^ OSM treatment triggers the expression of multiple antiviral genes that are involved in antigen processing or presentation and in immune regulation. In this regard, the findings of Ye et al^6^ generally are consistent with this trend and might reveal novel strategies for the development of new host-targeting, anti-HBV agents that resemble IFN-α.

Although the results presented by Ye et al are interesting, they should be interpreted with caution and multiple lines of additional investigations are required to address the remaining questions. First, OSM is a proinflammatory cytokine that might contribute to hepatic inflammation,^10^ it therefore will be of particularly great interest to determine whether administration of OSM might exacerbate pathogenic inflammation in HBV-infected liver and how this could be prevented. In relation to this, the safety profile of OSM as a foe-turned friend in the treatment of chronic hepatitis B might be a concern because it has been shown to be involved in various inflammatory diseases including arthritis, inflammatory bowel disease, asthma, dermatitis, and cardiovascular disease.^10^ Second, it will be of paramount importance to validate the safety and anti-HBV efficacy of OSM in physiologically relevant cultured cells and animals before it could be moved forward to human clinical trials. Although human liver organoids and primary human hepatocytes infected with HBV are preferred, even infected HepG2-NTCP cells are better than the HepG2-2.15 cells heavily used in the current study. Third, further mechanistic studies in several important areas are warranted to clarify how OSM inhibits HBV replication. Because both OSM and IFITM1 are IFN-stimulated genes,^11^ they are plausible effectors of IFN-α treatment. A side-by-side comparison of the therapeutic effect of IFN-α and OSM might be helpful. In addition, it will be crucial to clarify whether the nonspecific proinflammatory response induced by OSM contributes to its anti-HBV effect and whether OSM might have a direct or indirect impact on cccDNA. Because the antiviral activity of IFITM1 is thought to be mediated primarily through inhibition of viral entry,^12^ the influence of OSM and IFITM1 on HBV entry also should be investigated. We note that Figure 8 in the article by Ye et al shows the requirement of IFITM1 for the anti-HBV activity of OSM. However, the anti-HBV effects of OSM still were seen when IFITM1 was knocked out, implicating a role for other unknown genes. Thus, it will be intriguing to identify these genes and to clarify how they contribute to inhibition of HBV replication by OSM. Fourth, a combination of OSM with nucleoside analogs or IFN-α should be tested for synergistic or additive anti-HBV effects. Finally, whether IL6 or other cytokines in the IL6 family also might inhibit HBV replication through a similar mechanism merits clarification.

Host-targeting agents exert their anti-HBV effects through inhibition of HBV-dependency factors or activation of HBV-restriction factors. It remains controversial as to whether any of the host-targeting agents might directly target cccDNA, the major barrier to functional cure or complete cure. In principle, cccDNA-targeting agents could either act indirectly to counteract viral and cellular proteins required for cccDNA assembly and maintenance, or exert a direct effect on cccDNA, leading to cleavage and degradation.^13^ Some of the other host-targeting agents such as IL6 and OSM might be double-edged swords that inhibit HBV replication but simultaneously exacerbate pathogenic inflammation of the liver. Turning these foes into friends in the treatment of chronic hepatitis B might require extra effort to bring inflammation under control. Achieving a functional cure of chronic hepatitis B is a very challenging goal owing to cccDNA persistence. New efforts including a further test of OSM and other cytokines should be encouraged until better anti-HBV therapeutics are developed that bring us much closer to a functional cure.
